# The Death of Dr. W. L. Bowdoin

**Published:** 1870-08

**Authors:** 


### ARTICLE III.

### *The Death of Dr. W. L. Bowdoin.*

At a meeting of the American Academy of Dental Science, held in Boston, June 7th 1870, the following resolutions were unanimously adopted:

Whereas, The "American Academy of Dental Science" has received the sad intelligence of the sudden and unexpected decease of our esteemed friend and associate, Dr. W. L. Bowdoin, of Salem, Mass., therefore,

Resolved, That we have heard with the deepest sorrow of the death of our fellow member, Doctor Willard L. Bowdoin, who has departed in the prime of manhood, in the fullness of his usefulness and professional success.

Resolved, That while we bow in reverent submission to the will of Almighty God in taking from us our beloved friend, we deeply deplore the great loss we have sustained in his death.

Resolved, That by his death, the profession of dentistry, to which he devoted so many years of his life, has lost one of its brightest ornaments, the community in which he lived has been deprived of the valuable services of a skilful practitioner, and our society has been bereft of an highly esteemed member.

Resolved, That we his fellow members of the Academy, to whom he was greatly endeared, will affectionately cherish his memory, and will ever retain in happy remembrance his bright Christian example, his high reputation for honor, integrity, and professional courtesy, his retiring and genial disposition, his kindness of heart, and the many other virtues which adorned his character.

Resolved, That we tender to the family of Dr. Bowdoin, the assurance of our deepest sympathy in their heavy bereavment and affliction, and our heartfelt expression of sorrow and condolence.

Resolved, that these resolutions be entered upon the records of the Academy, and a copy of them be presented to the family of the deceased and that they be published in the American Journal of Dental Science, the Dental Cosmos, and Salem papers.

